# ‘Calibrating to scale: a framework for humanitarian health organizations to anticipate, prevent, prepare for and manage climate-related health risks’

**DOI:** 10.1186/s12992-020-00582-3

**Published:** 2020-06-26

**Authors:** Patricia Nayna Schwerdtle, Elizabeth Irvine, Sonia Brockington, Carol Devine, Maria Guevara, Kathryn J. Bowen

**Affiliations:** 1 Heidelberg Institute of Global Health, Heidelberg University, Heidelberg, Germany; 2 Nursing & Midwifery, Faculty of Medicine, Nursing & Health Science, Monash University, Melbourne, Australia; 3 Centre for Humanitarian Leadership, Deakin University, Melbourne, Australia; 4 Medicines Sans Frontieres, Geneva, Switzerland; 5 Medicines Sans Frontieres, Toronto, Canada; 6 Dahdaleh Institute for Global Health Research, York University, Toronto, Canada; 7 Fenner School of Environment and Society & Research School of Population Health, Australian National University, Australian Capital Territory, Australia. Institute for Advanced Sustainability Studies, Potsdam, Germany

**Keywords:** Humanitarian, Health systems, Climate change, Resilience, Operational framework

## Abstract

Climate Change is adversely affecting health by increasing human vulnerability and exposure to climate-related stresses. Climate change impacts human health both directly and indirectly, through extreme weather events, changing distribution of health risks, increased risks of undernutrition, population displacement, and greater risks of injuries, disease, and death (Ebi, K., Campbell-Lendrum, D., & Wyns, A. The 1. 5 health report. WHO. 2018). This risk amplification is likely to increase the need for humanitarian support. Recent projections indicate that under a business as usual scenario of sustained greenhouse gas emissions, climate change could double the demand for humanitarian assistance by 2050 (World Health Organization. Operational Framework for building climateresilient health systems. WHO. 2015). Humanitarian assistance is currently not meeting the existing needs, therefore, any additional burden is likely to be highly challenging.

Global health advocates, researchers, and policymakers are calling for urgent action on climate change, yet there is little clarity on what that action practically entails for humanitarian organizations. While some humanitarian organizations may consider themselves well designed to respond, climate change as a transversal threat requires the incorporation of a resilience approach to humanitarian action and policy responses.

By bringing together authors from two historically disparate fields - climate change and health, and humanitarian assistance – this paper aims to increase the capacity of humanitarian organizations to protect health in an unstable climate by presenting an adapted framework. We adapted the WHO operational framework for climate-resilient health systems for humanitarian organizations and present concrete case studies to demonstrate how the framework can be implemented. Rather than suggest a re-design of humanitarian operations we recommend the application of a climate-lens to humanitarian activities, or what is also referred to as mainstreaming climate and health concerns into policies and programs. The framework serves as a starting point to encourage further dialogue, and to strengthen collaboration within, between, and beyond humanitarian organizations.

## Background

Climate change is likely to increase humanitarian caseloads. The diverse group of actors providing humanitarian assistance locally, nationally and transnationally is increasingly aware of the impacts of accelerating climate change on vulnerable communities and paying more attention to climate change in their responses [1]. Climate change exacerbates existing health challenges - disease-related, food and water insecurity, mental health issues - and compromises human security and economic growth. [2, 3] - and also exacerbates existing health inequities, disproportionately affecting disadvantaged people [4]. There is a recognition that climate change has transitioned from an environmental issue to one with consequences for human health, human rights, equity, and social justice - which goes to the core of humanitarian principles; humanity, neutrality, independence, and impartiality. Under pessimistic scenarios with slow and unequal growth, the demand for external humanitarian assistance could double by 2050 however, there is still time to mitigate the worst effects of climate change on vulnerable populations [5] and leverage the social, economic, and health co-benefits of mitigation.

To date, humanitarian efforts to mitigate,^1^ adapt,^2^ communicate, and advocate concerning climate change are diverse and lack a systematic and collaborative approach. Whilst some humanitarian organizations debate if and where to begin, others are deeply engaged in climate and humanitarian issues from mitigation and adaptation, to loss and damage and geoengineering [6]. Notwithstanding the conceptual and logistical challenges, there is a need for strategic leadership from humanitarian governance as well as technical support and dedicated resources to more deliberately address climate change. Yet few frameworks, guidelines, and tools exist that can be used across the sector to move this action forward. The scale of humanitarian needs anticipated under all climate change scenarios will require unprecedented collaboration within the humanitarian community and beyond.

This paper aims to further catalyze these discussions and collaborations and increase capacity for protecting health in an unstable climate by presenting an operational framework for climate-resilient humanitarian organizations adapted from the widely accepted WHO model [7]. By implementing this framework, humanitarian health organizations may be better equipped to anticipate, prevent, prepare for, and manage climate-related health risks.

### An operational framework for climate resilient health systems adapted for humanitarian health organisations

The WHO operational framework for building climate-resilient health systems [5] starts with the building blocks of health systems many of which are shared by humanitarian health organizations such as; leadership and governance, health workforce, and service delivery. This framework was chosen for this commentary due to its practicality, that appeals to humanitarians and its broad application to a range of settings by WHO and member states. This original framework responds to a demand for guidance on how the health sector can systematically and effectively address the challenges of short term climate variability and long term climate change. By implementing the framework, health systems may be better equipped to anticipate, prevent, prepare for, and manage climate-related health risks.

**Fig. 1:**
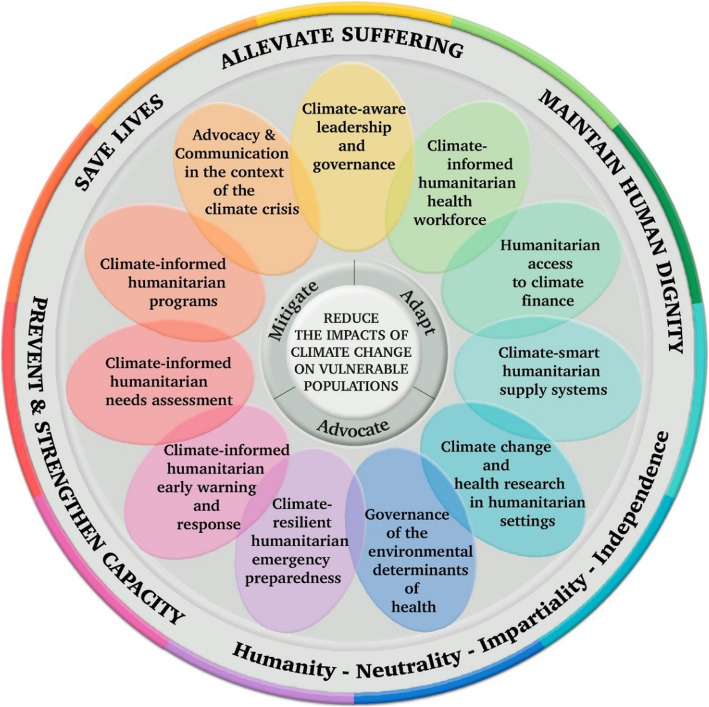
An Operational Framework for Building Climate Resilient Humanitarian Health Systems. Adapted from the WHO Operational Framework for climate-resilient health systems [7]. At the center: the core objective of the framework to reduce the impacts of climate change on vulnerable people and communities. Three main recognized climate actions in which humanitarians engage: Mitigation (to reduce contributions to climate change); Adaptation (to adjust to actual or expected effects of climate change by minimizing harm and to improve response effectiveness); Advocacy (including communications: to raise awareness about the humanitarian impacts of climate change, and to speak out on what is witnessed, amplifying voices of affected populations) [8]. Eleven elements of climate resilience in humanitarian terms explained below with examples. This rethinking of current humanitarian operations occurs in the context of broader humanitarian principles in the outer rim; Humanity, Neutrality, Impartiality and Independence, and the goals of humanitarian action; To save lives, alleviate suffering, maintain human dignity and prevent and strengthen capacity. The 11 elements of a climate-resilient humanitarian health organization

The original framework has been adapted (See Fig. 1) by firstly, translating the original ten elements into humanitarian terms and secondly, adding one additional element: communications and advocacy. Firstly, a humanitarian lens was applied to interpret each element into humanitarian terms for example, ‘vulnerability and capacity assessment’ becomes ‘humanitarian needs assessments’ and ‘essential medical products and technologies’ becomes ‘humanitarian supply’. Secondly, we add an eleventh element; ‘communication and advocacy’ due to its central role in humanitarian work. Case studies were purposefully selected by the authors as those most closely suited to the element described and as good examples of humanitarian adaptation.

### Climate-aware leadership and governance

Climate-aware leadership and governance entails that leaders consider climate risks in strategic planning over the short, medium, and long-term in addition to the core functions of ensuring good governance, accountability, and evidence-based policy within humanitarian organizations [9]. Along with diversity, inclusion, and socially responsible governance considerations, climate-aware humanitarian governance examines climate and environmental risk as part of their duty of care responsibility to the workforce, their ethical duty to ‘first do no harm’, and to improve safety and quality of care for patients.

Climate-informed leadership involves the development of clear mitigation and adaptation targets and indicators to enable the tracking of progress. These considerations can be framed in strategic and annual planning cycles (3–4 years), adapted to local contexts, and linked to longer-term climate science time frames (2030, 2050, and 2100). Governance bodies can strategically prioritize climate change internally and develop a clear vision for the organization whilst considering external positioning and influence, such as contributing to global climate discourse at the United Nations Framework Convention on Climate Change (UNFCCC) Conference of Parties. Strategic partnerships can be considered with government and other actors so that as the organization builds its capacity to be climate-resilient, it can support partners to do the same.

Case Study 1: Climate Leadership – The Red Cross Red Crescent Climate Centre

**Table:** 

**Who:** The Climate Centre was established in 2002 by the Netherlands Red Cross and the IFRC and offers strategically important knowledge, specialist services, and advice on best practice. **What:** The overarching mission of the Climate Centre is to help the Red Cross and Red Crescent Movement and its partners reduce the impacts of climate change on vulnerable people. Yet they have undoubtedly stretched beyond this objective and act as a resource for the entire sector with their frameworks, open-access teaching, and learning materials, publications, and case studies of climate change adaptation. **How:** The center aims to: Raise awareness on climate change; Provide humanitarian assistance; Improve response and preparedness; Decrease vulnerability of communities; Integrate climate risk management into policy and planning and; Mobilize human and financial resources.	

### Climate-informed humanitarian health workforce

Humanitarian health professionals possess individual skill-specific competencies such as those obtained by nursing, medical or allied health degrees, and also shared core humanitarian competencies [10]. This subgroup of ‘human resources for health’ includes all people engaged in actions whose primary intent is to enhance health in humanitarian settings [11]. As awareness of the health risks of climate change is essential for strengthening health systems against climate change, the disciplines from which humanitarian health professionals originate are gradually integrating sustainable health care into curricula [12, 13]. Humanitarian health organizations will benefit from future health professionals becoming more aware of planetary health issues, however, in the meantime, they need to equip the existing workforce with the knowledge, skills and attitudes to practice effectively during the climate crisis.

A climate-resilient humanitarian health workforce would be able to practically link climate change and health, be aware of changing disease profiles in response to climate change and effectively communicate with the public and decision-makers to advocate for climate action [14, 15]. They would understand that the health sector contributes significantly to emissions and environmental degradation, and implement mitigation measures that have co-benefits for health [16]. Finally, they would have the skills to engage with and empower affected communities to take ownership of their response to health challenges amplified by climate change. Together these workforce competencies would build organizational capacity to respond effectively and with flexibility to climate change [17].

Case Study 2 Humanitarian relevant e-learning on climate change and health

**Who:** Oxford University and the Chinese University for Hong Kong (Disaster and Medical Humanitarian Response) offer a particularly relevant online course to humanitarian health workers that is available on the prevention web platform. Similar courses are available from the Humanitarian Leadership Academy, Red Cross Red Crescent Climate Centre, and the United Nations Climate Change Learning Partnership.

**What:** The course entitled ‘Climate Change and Health’ targets individuals studying and working in health, policy, education, and humanitarian sectors and is suitable for healthcare personnel and frontline disaster relief practitioners.

**How:** Open, self-paced and free to any learner worldwide, the course introduces important concepts of how to tackle climate change-related health impacts and how to support community preparedness, response, policy formulation, and implementation.

### Humanitarian access to climate finance

More people than ever need humanitarian assistance, yet the gap between requirements and funding has never been greater [18]. Although the proportion of total climate adaptation funding being spent on health is slowly increasing, there are concerns that climate finance is not reaching the most vulnerable and those with the least capacity to adapt. These factors represent ‘a perfect storm’ for humanitarian finance in a competitive and shrinking fundraising space, affected by changing solidarity patterns, the global COVID19 pandemic and competing domestic priorities.

To increase eligibility for climate finance, humanitarian health organizations need to have clear internal positioning on climate change, evidence of mainstreaming climate change into policies and programs, and a record of accomplishment of mitigation and adaptation activities. There are numerous ways to engage with the major climate change funding mechanisms, including the Global Environmental Facility (GEF) and the Green Climate Fund (GCF), such as becoming accredited or becoming a delivery partner of an accredited agency. Green bonds or climate bonds could also represent a new funding stream for humanitarian organizations to mitigate and adapt their activities. There is a need to engage in and develop new humanitarian finance models that leverage climate funding streams whilst upholding the humanitarian principle of independence: that action must be autonomous from the political, economic, or military objectives of other actors.

Case Study 3 Save the Children – Green Climate Fund Accreditation

**Who:** Save the Children Australia is the first non-environmental, non-governmental humanitarian organization to be accredited by the Green Climate Fund and is accredited on behalf of the global Save the Children movement.

**What:** Accreditation will allow Save the Children to collaborate with countries hardest hit by climate change and apply for funding from the USD$10 billion funds. Save the Children will work with national governments and local communities most severely impacted, but not able to access the GCF independently to develop and deliver programs that respond to climate change.

**How:** Save the Children has an established history of climate change programming and will leverage its significant global network of specialist technical advisors working in climate change, health, nutrition, WaSH, livelihoods, agriculture, and food security to develop and deliver programs.

### Climate-smart humanitarian supply systems

Health supply systems ensure access to essential medicines and equipment to provide safe and cost-effective healthcare interventions. For humanitarian health organizations, this occurs in some of the world’s most challenging contexts, often existing at the edge of climate extremes. For climate resilience, humanitarian organizations face two additional considerations: firstly, how climate threats could compromise or rupture the supply chain and secondly, the sustainability of supply from procurement to end-of-use management.

Humanitarian organizations have an opportunity to embrace ‘smart, safe and green’ health supply chains and infrastructure options that aim to be low carbon, have minimal impact on the environment, save money and be resilient to natural hazards [14]. Indeed, many organizations are piloting, implementing, and scaling renewable energy in health facilities.

Whilst procurement often involves quality and cost considerations, environmental impact, and ethical sourcing of materials and processing is rarely integrated into selection criteria. Sustainability of the procurement process includes consideration of embedded carbon of items, transport, and disposal as well as beyond emissions, to the environmental impact of the item from manufacture to disposal. Inherent within these considerations is an assessment of the life cycle of health care products and whether sourcing locally could mean reduced emissions and increased efficiency whilst maintaining quality. Certainly, during COVID19 localized supply chains are being viewed in a new light. When considering sustainable supply there is a need for solid evidence to pull on the right levers that save money and carbon without compromising quality assurance. Such levers may involve improving air: sea freight ratios, enabling and empowering locally hired staff to reduce the need for headquarter-field visits.

Case study 4 Médecins Sans Frontières project measuring the carbon footprint and environmental impact of humanitarian operations

**Who:** The Transformational Investment Capacity (TIC) is a fund that supports MSF to find new solutions in addressing the humanitarian healthcare needs of vulnerable populations around the world.

**What:** This simple environment impact toolkit and guidance framework help MSF offices and operations systematically measure their major environmental impacts. The tool measures energy use, transport, and waste and offers mitigation suggestions. The goal is not to benchmark activities but to gain insights into the key environmental impacts and to identify opportunities to mitigate them.

**How:** MSF partnered with sustainability experts and other organizations with expertise in carbon mitigation and waste management, such as ICRC and Global Green and Healthy Hospitals.

### Climate change and health research in humanitarian settings

Climate change and health research uncover the pathways by which global warming, directly and indirectly, influences human health and explores the health co-benefits of mitigation and the effectiveness of adaptive responses. A robust flow of knowledge about new patterns of hazard and vulnerability is essential to enable efficient allocation of scarce resources and to make health-related progress against current and emerging climate threats [19].

Whilst evidence on climate change and health date back over thirty years, there are significant gaps especially concerning the most vulnerable populations, with the least capacity to adapt [20]. A synergistic opportunity, therefore, lies with humanitarian organizations to collaborate with research institutions to develop applied research programs aimed at strengthening climate-informed health care and humanitarian responses. Whilst most humanitarian organizations have data-sharing agreements, few partnerships maximize this opportunity. Further, no comprehensive research strategy outlines the important climate change and health gaps humanitarians could fill. For example, most heat health research pertains to high-income countries and there is a paucity of research on how heat affects vulnerable populations in refugee camps and urban slums [21, 22]. Researchers need to realize that humanitarians are often looking for time-critical operational insights that strengthen responses and benefit communities. Similarly, humanitarian actors should be aware that standard epidemiological methods and tools are ill-equipped to answer climate change-related questions dealing with long timeframes across broad geographical areas.

Case study 5 An academic research partnership-linking climate with humanitarian assistance

**Who:** The International Research Institute for Climate and Society (IRI) has partnered with several humanitarian organizations including the Red Cross/Red Crescent and Oxfam to increase humanitarian capacity to manage changing climate risks.

**What:** The mission of the IRI is to enhance society’s capability to understand, anticipate, and manage the impacts of climate to improve human health and wellbeing and the environment, especially in developing countries. The IRI engages in strategic and applied research, education, capacity building, and by providing forecasts and information products with an emphasis on practical and verifiable utility and partnership.

**How**: The partnership between IRI and the Red Cross/Red Crescent comprises online map rooms, IRI graduate student internships, and a help desk through which climate scientists provide rapid responses to questions from humanitarians on matters related to forecasts, weather, and climate.

### Governance of the environmental determinants of health

Climate change threatens health through environmental determinants that include air, water, soil, food, and the built environment. Although health outcomes are also mediated through socio-economic factors the most effective humanitarian actions promote the safety and security of environmental determinants [9].

Minimum standards for humanitarian activities provide guidance on incorporating environmental considerations into responses, however, the added pressure climate change exerts and the increasingly protracted nature of humanitarian responses means more attention needs to be paid to the direct effects of the environment on health in operational settings [9]. Some practical considerations may include:

- **Air quality:** Consider distributions of safe and energy-efficient heating and cooking in humanitarian settings. Indoor air pollution from cooking sources is a major cause of respiratory-related morbidity and open fires increase the risk of injury and death [23].
- **Water quantity and quality:** Implement climate-resilient water safety plans in both rural and urban contexts. Define and monitor water quality standards and evaluate access to water in the context of water insecurity [9].
- **Food and nutrition security:** Climate change affects food security in terms of both quantity and nutritional quality [7]. This requires consideration when planning responses to both protracted nutritional crises and emergencies. Consider linking weather forecasts and climate data with local knowledge (from farmers and communities) to estimate and project food insecurity. Conduct nutritional assessments integrating climate and weather information, provide pre-emptive supplementary feeding when indicated whilst considering vaccination and prophylactic health care interventions to vulnerable groups [3].
- **Shelter:** Consider whether the shelter is equipped to deal with projected changes in weather and climate such as extreme heat, flood, and storm risk. Consider ventilation standards, sustainable building design, and clean energy. Combine organizational expertise with local community consultations to determine the most feasible, safe, and green designs. This may require concerted advocacy efforts for states who oppose permanent structures to avoid hosting refugees in the long term [23].
- **Waste management:** Waste prevention and minimization, identify products that can be avoided or minimized upstream, safer disposal, and connect field operations to existing recycling programs. Innovative thinking and localization are critical, asking communities what they need and co-designing assistance to minimize waste, which may include providing cash aid instead of items [23].

Case study 6 BRAC humanitarian interventions prioritizing the environmental determinants of health

**Who:** BRAC is one of the largest NGOs in the world. BRAC’s mission is to alleviate poverty and encourage economic participation by empowering people through social and economic programs.

**What:** As a component of their Climate Change Program, BRAC addresses mitigation, adaptation, and air pollution reduction efforts together with agroforestry and block plantations [24].

**How:** Bangladesh is among the countries with the worst air quality worldwide, with about 21% of deaths attributed to air pollution nationally [25]. Vegetation can play an important role in tackling the dual challenge of climate change and air pollution as natural vegetation can remove a range of pollutants in the air, and mitigate climate change through carbon sequestration [7]. Planting in environmentally critical areas, such as those prone to droughts, cyclones, and floods, serves multiple benefits including adaptation through minimizing environmental degradation and in humanitarian and development contexts, enhancing livelihood options.

### Climate-resilient humanitarian emergency preparedness

Disaster response preparedness commonly known in the aid sector as ‘emergency preparedness’ (E-Prep) refers to pre-disaster activities undertaken to minimize disaster-related injury, loss of life and property damage, and to ensure that rescue, relief and rehabilitation services can be provided in a timely and appropriate manner following a disaster.

In developing humanitarian E-Prep that is climate-resilient, emergency and disaster risk management protocols and policies should be informed by current and likely future climatic conditions. As climate change makes crises more unpredictable and intense there will likely be a need to adopt more regional approaches and pre-position health and relief goods. Sourcing E-Prep locally and regionally has proven important during the COVID19 pandemic, with lessons learned that can be applied in future climate-related disasters.

Predictive analytics can help identify where the worst impacts meet the most vulnerable and exposed populations, lessening uncertainty to some degree, and enabling better preparation. Investing in collaborative processes and tools such as predictive modeling, or machine learning (See AIME case study below) organizations can ensure activities are climate-smart. It will be important to ensure E-Prep assets (materials and tools) and activities are more resilient to climate hazards, such as non-plastic shelters, community-based early-warning systems, and advanced health informatics.^3^

Case study 7 AIME – Artificial Intelligence in Medical Epidemiology

**Who:** Forecast information has been used by governmental and non-governmental organizations in Malaysia and Brazil to predict with precision where outbreaks of Dengue and Zika are likely to occur.

**What:** AIME’s flagship product - AiRBO- (AI-Console for Arboviral Diseases) is an AI-capable, web-based Geographic Information System (GIS) equipped not only for ongoing Dengue outbreak surveillance, and enhanced analytics but also with predictive capability for informing users where and when the next Dengue outbreak will likely take place, as early as 30 days in advance. Its predictive algorithm has also been expanded to encompass other arboviral diseases, such as Zika and Chikungunya.

**How:** Where multiple outbreaks take place simultaneously, AIME creates a prioritization index across the affected region through a predictive algorithm. The algorithm interposes multiple variables including epidemiologic data, geographic landscape, meteorological information, and wind direction. This aids in the identification of priority areas where insecticide, larvicide, and human resources should be deployed first.

### Climate-informed humanitarian early warning and response

Early warning systems (EWS) are a means by which people systematically receive relevant and timely information before a disaster to make informed decisions and take action. There are many existing EWS that humanitarians utilize such as the Humanitarian Early Warning Service, the FAO Global Information and Early Warning System, the Forum on Early Warning and Early Response, the Famine Early Warning System, and the Global Disaster Alert and Coordination System.

Climate-resilient humanitarian aid workers would routinely utilize climate-informed EWS, meaning they would integrate weather and climate information (e.g – hazard maps) for more accurate situational analysis in the context of a rapidly changing climate. To become climate-informed and weather aware, humanitarian organizations might review their disease surveillance indicators and the early warning systems they utilize, investigate the extent to which their information system integrates weather and climate data, and connect with organizations that might strengthen the validity and reliability of monitoring tools. This would allow more accurate targeting of interventions including communication with communities. It is important to connect EWS with actual responses and ensure there is surge capacity when needed. Ideally, these systems would be used to their full capacity across extreme weather events, modeling of climate-sensitive disease outbreaks, and for slower onset impacts like drought and sea-level rise [26].

Case Study 8 IFRC Forecast based financing

**Who**: One of the financial mechanisms available to fund the Red Cross Red Crescent National Societies’ ‘Early Action Protocols’ activities is Forecast based Action (FbA) by the Disaster Relief Emergency Fund.

**What**: The goal is to anticipate disasters, prevent their impact, if possible, and reduce human suffering and losses. FbA enables access to humanitarian funding for early action that can be taken based on meteorological forecast information, combined with risk analysis, to prepare for extreme weather events.

**How**: There are three components: **1. Triggers**: Danger levels are identified for a region based on detailed risk analysis of relevant natural hazards, impact assessments, and vulnerability data. A forecast trigger then gives notice before the danger level is reached. **2. Selection of actions:** The pre-defined actions to be implemented at the time of a triggering forecast to reduce the humanitarian impact of an event. **3. Financing mechanism:** An ex-ante financing instrument that automatically allocates funding once a forecast is triggered, thus enabling effective implementation of early actions. ‘Early Action Protocols’ serve as action guidelines delineating roles and responsibilities for quick action when a trigger is reached [5].

### Climate-informed humanitarian needs assessment

Humanitarian needs assessments use various methods to collect and analyze information to determine what assistance is needed, identify individuals and groups most at risk and to make good decisions for allocating resources to meet the needs of disaster-affected communities. In this way, the humanitarian principle of impartiality is realized. Complemented by wider situational analysis (safety and security) and specific health assessments and environmental analysis, robust needs assessments are a critical component of effective humanitarian response [3].

Humanitarian needs assessments can be more climate-informed by including a review of the latest vulnerability and adaptation assessment or the Health National Adaptation Plan (H-NAP) as a component of their contextual assessment. Other useful resources include the UNFCCC - WHO Health and Climate Change Country Profiles that documents priority climate change and health impacts, how they are projected to develop according to different climate scenarios, and what actions are being planned or undertaken to address them [27].

The short, medium and long-term environmental impact of humanitarian activities would ideally be included in needs assessments. Additionally, humanitarians might reflect on how drivers of environmental change (such as climate change, demographic shifts, and urbanization) affect ecosystem services and human health. More specific needs assessment indicators might also consider the key climate change and health risks in the setting and whether people are trapped within or displaced into more climate-vulnerable areas such as low lying coastal urban slums or landslide-prone areas, putting them at risk of different climate-related hazards and possibly secondary displacement.

Finally, whilst traditional humanitarian needs assessment tools tend to be reactive and implemented post-disaster, a climate-resilient needs assessment tool might be more pre-emptive, allowing the delivery of humanitarian aid pre-hazard to allay disaster, for example, forecast-based finance (See Case Study 8). This is a good example of how climate change adaptation can be an opportunity for the humanitarian, development, and peace-building nexus to collaborate by building local capacity to prepare, respond, and rebuild post-disaster.

Case Study 9 Assessing the needs of people and their environments – EHA Connect

**Who:** The Environmental Humanitarian Action (EHA) Connect platform joins the humanitarian and environmental communities together to support environmentally sustainable disaster management.

**What**: This platform helps humanitarians develop more resilient emergency management systems and assists environmental actors to engage in the disaster space.

**How:** On the EHA platform, the United National Environmental Program has developed an environmental needs assessment in post-disaster situations practice guide. This guide is intended for use by anyone concerned with environmental and related impacts occurring in a post-disaster situation. This guideline can be combined with organization-specific tools to consider both human needs and environmental needs to reduce the environmental impact of humanitarian activities.

### Climate-informed humanitarian programs

Humanitarian crises are becoming increasingly protracted with the average duration increasing since 2005 from four to seven years in 2017 [28]. Extreme weather events are now being described as Compound (combining extreme heat and dryness affecting wildfire risk, air pollution, and food production); Cascading (successive climate hazards deprive societies of the time needed to recover in between), and Connected across continents [29]. These factors suggest humanitarians plan longer-term interventions, incorporate climate considerations in planning and shift their goals from short-term stability towards longer-term resilience.

Meteorological and hydrological services should ideally work together with health services to monitor and prepare for climate-related risks to health. Considering there are roughly 80 climate-sensitive diseases [20] humanitarian programs could integrate climate change considerations into existing programs to strengthen disease control. New dialogues between national meteorological services where they exist and humanitarians can help enhance the provision of targeted and actionable climate information and operational early warnings for specific health applications [19]. Community engagement is critical for climate-informed humanitarian programs where nature-based solutions can be identified by incorporating indigenous and local knowledge into humanitarian responses.

Beyond traditional partnerships, humanitarian organizations might consider non-traditional partners beyond academia such as environmental organizations and architectural institutes that specialize in green infrastructure. Programs can be more climate literate by utilizing the UNFCCC-WHO country climate profiles (See Element 9) in planning and consider reviewing National Adaptation Plans, which should have a health component (H-NAP).

Case Study 10 Adaptation to Climate Change in Sub-Saharan African Humanitarian Situations

**Who:** This climate change adaptation in humanitarian settings project involves the United Nations World Food Programme, the UN Environment/OCHA Joint Unit (JEU), UNHCR, UNICEF, and the World Wind Energy Association (WWEA).**What:** The ‘Adaptation to Climate Change in Sub-Saharan African Humanitarian Situations’ project aims to strengthen climate change adaptation in targeted vulnerable humanitarian settings. The project supports internally displaced people, refugees, and host communities facing climate-related risks in Sudan, Burundi, and Chad.

**How:** The main outputs of the project are: 1. Improved understanding and integration of climate-environment risk planning and preparedness processes and tools. 2. A ‘No regrets strategy’ integrated into existing humanitarian programs, focusing on improving access to renewable energy, reforestation, and water management. 3. Individuals, communities, and governments in the target sites have access to knowledge, skills, and tools to address the impacts of climate change [30].

### Humanitarian communications and advocacy in the context of climate change

This eleventh element of the framework is specific to humanitarian organizations, especially those with a mandate to witness and consider speaking out about human suffering in crisis.

The magnitude and severity of climate change impacts on humanitarian needs will be determined by global action taken to reduce emissions and expedite action to cope with its effects [31, 32]. Therefore, advocacy and communications are tools humanitarians can exercise to respond to humanitarian crises and also to prevent them. Humanitarian organizations will have a limited ability to respond to the enormous needs projected, even with urgent mitigation. As such, targeted climate advocacy on health may be an impactful contribution to prevention. The next nine years are particularly critical [2] further building the argument for humanitarians to increase public awareness about the connections between climate and humanitarian health crises and urge citizens, industry, and governments to be responsible and accountable. Humanitarian voices are being called for to advocate for vulnerable people during high-level climate discourse, whether related to loss and damage negotiations, protecting climate-migrants, or geoengineering that has been described as a humanitarian concern [6].

Humanitarian organizations have the proximity to witness suffering and loss of life exacerbated by climate change and other factors. Therefore, they can amplify the voices of those most affected and least responsible. Similarly, health professionals have a key role to play in communicating the health risks and driving a robust response to improve human health [4]. This is further supported by health professional ethical codes and professional standards requiring them to investigate the causes of ill health and either address them directly or advocate that they are tackled.

A climate-resilient humanitarian health organization would be active in climate change and health responses to be in a credible position to raise awareness and advocate for climate justice. Further, they would have strong internal positioning to allow external engagement. Such an organization would influence the public and decision-makers to change policies, practices, and systems towards renewables, divestment in fossil fuels, and a more sustainable, equitable future.

Case Study 11: CARE advocacy at the IPCC Conference of Parties

**Who:** CARE is a major international humanitarian agency delivering emergency relief and long-term international development projects. CARE is non-sectarian, impartial, and non-governmental and one of the largest and oldest humanitarian aid organizations focused on fighting global poverty.

**What:** CARE’s overarching objectives in their response to the climate emergency are to empower poor and marginalized people, particularly women and girls, to take action on the climate crisis at all levels and to build knowledge for global change.

**What:** CARE made four demands at COP25 [27]: **Ambition:** Commit to submitting advanced climate action plans by 2020 that support progress towards the 1.5-degree limit that boosts gender-responsive climate action. **Finance:** Developed countries, in particular, must significantly increase finance for gender-just climate-resilient measures. **Gender:** Approve a strengthened UNFCCC gender action framework. **Agriculture:** Agree on deliverables that demonstrate scaled-up early action for sustainable, gender-equitable, and resilient action.

## Conclusion

Climate change is described as the issue of our time, shaping the health of a child born today [4]. The climate crisis demands a transformational change of human systems and the humanitarian sector is not exempt. Humanitarian leaders need to set the strategic vision, provide technical assistance, and dedicate resources so they are better equipped to deliver quality services during the climate crisis. Responding adequately and effectively can be considered a matter of best practice and maintaining the quality of humanitarian health care. The operational framework presented in this review serves as a starting point to encourage further dialogue and to strengthen collaboration within, between, and beyond humanitarian organizations.

A climate-resilient humanitarian health organization is one that is aware of its emissions and waste practices and takes active and progressive steps to mitigate its impact on the environment. It deeply understands the impacts of climate change on the communities with whom it works and molds all systems, structures, partnerships, and programs with this in mind. Such an organization can mainstream climate-smart policies across humanitarian programs to enable the long-term vision for a rapid and effective response. With a foundation of clear internal positioning, such an organization has the authority to speak out and is willing to engage in global climate discourse to advocate for vulnerable populations.

## Data Availability

The literature review search strategy available upon request.

## Footnotes

1 Mitigation: A human intervention to reduce emissions or enhance the sinks of greenhouse gases.

2 Adaptation: The process of adjustment to actual or expected climate and its effects, in order to moderate harm or exploit beneficial opportunities.

3 Health informatics: Information engineering (informatics) applied to the field of health care, essentially the management and use of patient health care information.
